# Integrating Multiple Lines of Evidence to Explore Intraspecific Variability in a Rare Endemic Alpine Plant and Implications for Its Conservation

**DOI:** 10.3390/plants9091160

**Published:** 2020-09-08

**Authors:** Martino Adamo, Stefano Mammola, Virgile Noble, Marco Mucciarelli

**Affiliations:** 1 Dipartimento di Scienze della Vita e Biologia dei Sistemi, Università degli Studi di Torino, Viale Pier Andrea Mattioli, 25, 10125 Torino, Italy; marco.mucciarelli@unito.it; 2 Molecular Ecology Group (MEG), Water Research Institute (IRSA), National Research Council (CNR), Corso Tonolli, 50, 28922 Verbania, Italy; stefano.mammola@irsa.cnr.it; 3 Laboratory for Integrative Biodiversity Research (LIBRe), Finnish Museum of Natural History (LUOMUS), University of Helsinki, Pohjoinen Rautatiekatu 13, 00100 Helsinki, Finland; 4 Conservatoire Botanique National Méditerranéen, Avenue Gambetta 34, 83400 Hyères-les-palmiers, France; v.noble@cbnmed.fr

**Keywords:** Apennines, genetic diversity, leaf morphology, South-Western Alps, species distribution modelling, *Tephroseris balbisiana*

## Abstract

We studied the ecology, distribution, and phylogeography of *Tephroseris balbisiana*, a rare plant whose range is centered to the South-Western Alps. Our aim was to assess the extent of intraspecific variability within the nominal species and the conservation status of isolated populations. We studied genetic diversity across the whole species range. We analyzed leaf traits, which are distinctive morphological characters within the *Tephroseris* genus. A clear pattern of genetic variation was found among populations of *T. balbisiana,* which clustered according to their geographic position. On the contrary, there was a strong overlap in the morphological space of individuals across the species’ range, with few peripheral populations diverging in their leaf morphology. Studying habitat suitability by means of species distribution models, we observed that *T. balbisiana* range is primarily explained by solar radiation and precipitation seasonality. Environmental requirements could explain the genetic and morphological uniformity of *T. balbisiana* in its core distribution area and justify genetic, morphological, and ecological divergences found among the isolated populations of the Apennines. Our findings emphasize the need to account for the whole diversity of a species, comprising peripheral populations, in order to better estimate its status and to prioritize areas for its conservation.

## 1. Introduction

South-Western Alps are regarded as a global biodiversity hotspot, since they host the highest numbers of rare and endemic species within the entire Alpine chain [1,2,3,4,5]. This high diversity is often linked to the complex biogeographic history of this area, which was greatly influenced by glaciations and post-glacial events throughout the Quaternary period. Massive and repeated glaciations of the Pleistocene have shaped the composition of the flora, influencing species diversification. In reason of this complexity, developing effective monitoring and conservation programs for the flora requires that evolutionary events at the origin of this diversity are interpreted carefully.

First, several lines of evidence have shown that only a little proportion of the Alpine flora originated by in situ species diversification (only ca. 1.2% of the native and ca. 9% of the endemic flora of the Alps have originated in this mountain range; [6]). As reviewed by Kadereit [6], the flora of the Alps is intimately linked to neighboring mountain ranges, mainly the Pyrenees, Apennines, Carpathians, Dinarids, and Balkans where most of the actual alpine lineages found shelter during glaciations (ca. 33% of the endemic species of the Alps; [6]). For example, in *Senecio* (Asteraceae), which is one of the largest genus of flowering plants, four ecologically differentiated, genetically distinct, and partly reproductively isolated groups have been recently described for the Alps [7]. Among the four new species presently recognized, three have considerable large distributions going from the neighboring Spanish Sierra Nevada to the Carpathians and only one have apparently originated in the Alps.

Secondly, it is a matter of fact that for the flora of the Alps and other large mountain ranges, species richness only partly correlates to genetic diversity. In a milestone contribution to this subject, [8] demonstrated that while the highest species richness is found in the South-Western Alps, the greatest genetic diversity within and among species is located in the central and eastern part of the Alpine chain [9,10,11,12]. Environmental characteristics can influence taxonomic and genetic diversity in different ways, resulting in a negative correlation between these two features [13]. In mountain contexts without the strong impact presence of glacial refugia (e.g., Carpathian Mountains), genetic diversity and species richness do not seem to be clearly linked [14]. The reason for low genetic diversity is the small population sizes observed under refugial conditions [8]. During the climatic oscillations and particularly the glaciation cycles of the Pleistocene, several animals and plants have survived as sparse stands of small populations in refugial areas in the South-Western Alps [15,16,17]. Most of these species found shelter in high-stress habitats such as mountain screes and xerophilous grasslands, thus facing varying but significant selective pressures [2,18,19]. Genetic drift promoting random loss and fixation of alleles at these stands has conditioned the distribution of genetic diversity [8].

In this complex evolutionary and geographical context, species delimitation has crucial implications from a conservation point of view [20,21]. In fact, the adoption of measures to counteract extinctions of rare taxa should rely on a precise estimation of the amount of diversity and of its allocation between species, habitats and genes [22]. The Unified Species Concept formulated by de Queiroz [23] considers “separately evolving metapopulation lineages” as the only necessary criteria for the determination of the species category [23,24]. The recognition at different integrated levels (e.g., morphology, genetics, ecology) of such lineages is therefore fundamental [25].

*Tephroseris balbisiana* (Asteraceae) belongs to the alpine and subalpine hygrophilous tall herb communities known as “megaforbs”, a species-rich habitat which flourish at altitude as high as 2250 m only in the South-Western Alps, thanks also to warmer temperatures and more abundant precipitations than in the rest of the Alps [3,26]. *Tephroseris balbisiana*, as well as other European *Tephroseris* should be treated as an allogamous taxon without apomictic reproduction [27]. Dense populations of *T. balbisiana* can be encountered at the very border of Italy and France in the Mercatour-Argentera Massif in the French Department of Alpes-Maritimes and in the Ligurian and Maritime Alps in Piedmont (Italy) [19,28]. Nonetheless, a peculiar disjointed distribution has been proposed in the past for this plant. Second and third restricted peripheral ranges lie in the Cottian Alps on the slopes of mount Monviso and on the Ligurian Apennines north to Genoa in the Turchino Pass area [29,30]. More recently, a fourth putative population of *T. balbisiana* was found in Emilia Romagna (Bologna, Italy) not far from the boundary with Tuscany [31].

In the Alps, *T. balbisiana* can be regarded as a glacial relict and a habitat specialist. Thus, the presence of disjointed Apennine populations is difficult to interpret in the light of the ecology and the biogeographical history of these Alpine populations. In this study, by integrating multiple lines of evidence (genetic analyses, leaf morphological analyses, as well as species distribution modelling), we explored the extent to which peripheral populations differ from the populations in the core area in their genetic, morphological and ecological characteristics. The integration of these multiple approaches allowed us to identify the potential presence of underestimated diversity within the species: A finding that enhance to estimate conservation status of this taxon below the species level and the spatial prioritization of conservation actions.

## 2. Results

### 2.1. Genotyping by Fingerprinting

We used five SCoT markers to genotype 75 individuals from nine populations distributed along the whole *T. balbisiana* geographic range. We scored a total of 66 different loci from bands with sizes between 513bp and 3000bp base-pairs long. According to the *informloci* function, 64 out of 66 loci were informative. The number of markers and loci scored resulted statistically supported as shown by the genotype accumulation curve (Figure S1). The 75 individuals corresponded to 73 observed multilocus genotypes with a global H (genotypic diversity index) between genotypes of 4.30 (Table S1). SM is the population with the lowest H diversity (1.39), while PO showed the highest H diversity (2.48). AMOVA showed that the variation is lower among samples within populations (36.57%, Phi = 0.39) than within individuals (55.74%, Phi = 0.44), while only 7.69% (Phi = 0.077) of the variation is among populations (*p* < 0.05) (see Table 1).

**Figure 1:**
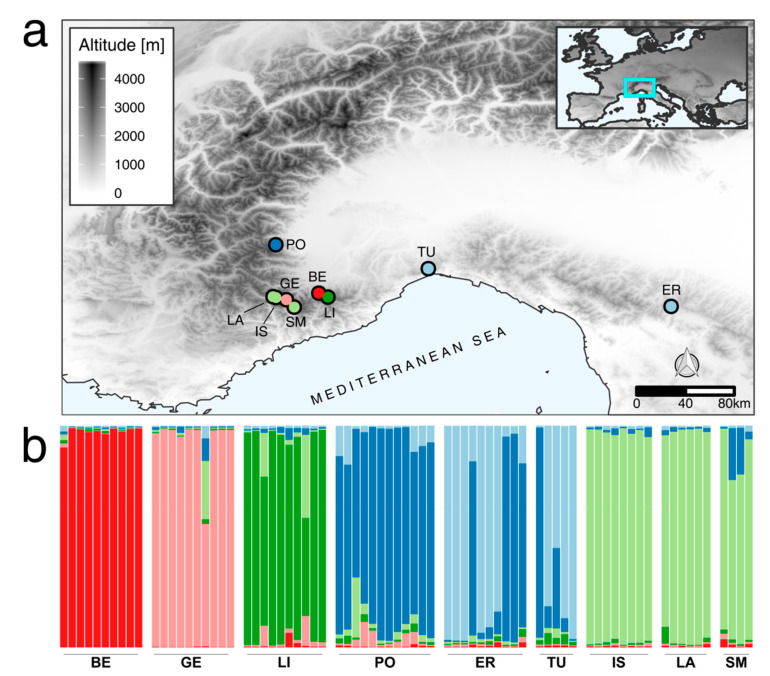
Geographic framework and STRUCTURE analysis results: (**a**) SCoTs genotyped populations across the species range. Background grey scale represents altitudes. Each dot corresponds to a studied population in its geographical position and dot colors correspond to the most probable cluster (K) retrieved by means of the STRUCTURE analysis. Dot color code and STRUCTURE analysis color code match. (**b**) results of the STRUCTURE analysis for K = 6 as determined with Structure Harvester in Evanno hypothesis. LA, IS, SM, and GE populations were from Maritime Alps; LI and BE were from Ligurian Alps; PO was from Cottian Alps and TU and ER were from Appennines. See Table 4 for populations details.

According to the Structure Harvester output based on STRUCTURE results, the K value associated with the modal ΔK was six (Figure 1b), thus corresponding to six genetic clusters. However, as shown in Figure 1b, only three of the genetic clusters were almost exclusive of specific populations (BE, GE, LI). In the populations PO, ER, and TU a mixed membership was evident. ER and TU populations showed the highest rate of admixture. Westernmost individuals grouped in a single cluster exclusive of these three populations (LA, IS, SM).

### 2.2. Phylogenetic Markers

**Figure 2:**
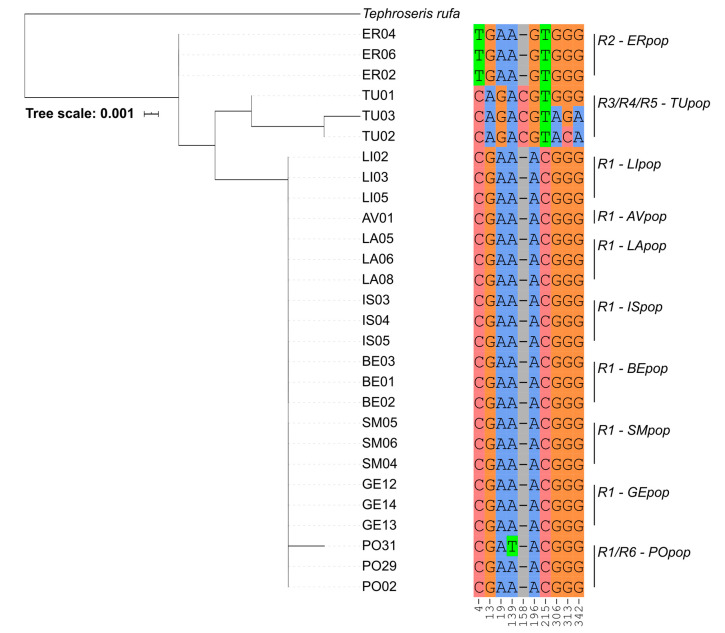
Ribotypes partial alignment of all the 28 analyzed samples: Each nucleotide corresponds to a specific position into the global alignment reported at the column base.

While we detected no nucleotide polymorphisms along plastidial sequences (trnL, psbB, and psbC), there were differences in the ITS sequences determining six different ribotypes (Figure 2). Four out of the six ribotypes were found in the Apennine populations ER and TU, with a strong differentiation in TU, for which each individual has its own variant (R3/R4/R5). All the other populations are represented by a single ribotype (R1) with the exception of a single sample belonging to the PO population, in which a further variant (R6) has been identified.

In the phylogenetic analysis (Figure S2) the ITS sequences of *T. balbasiana* positioned into three distinct groups corresponding to the different ribotypes obtained with the previous analysis (cf. Figure S2 with Figure 2). Sequences of *T. balbsiana* corresponding to R1 and R6 ribotypes grouped together in a large clade containing all the French and most of the Italian individuals together with *T. coincyi*. Conversely, individuals of ER population of Emilia Romagna belonging to the R2 ribotype shared a separate subclade with the sequences of *T. longifolia* subsp. *brachichaeta* (syn. *T. italica*). These latter belonged to four different populations having the same geographic origin of *T. balbisiana* of the Tuscan-Emilian Apennine. The Apennine population of the Turchino Pass (TU), instead, grouped in a larger clade with the *T. tenuifolia* aggregate of species: *T. longifolia* subsp. *longifolia*, *T. longifolia* subsp. *gaudinii*, *T. longifolia* subsp. *pseudocrispa* and *T. longifolia* subsp. *moravica* (Figure S2). The same clade contained *T. longifolia* subsp. *aucheri* and *T. papposa*. With the exclusion of the TU population, ribotypes and phylogeny of *T. balbisiana* were geographically coherent in Figure S4.

### 2.3. Leaf Morphology

**Figure 3:**
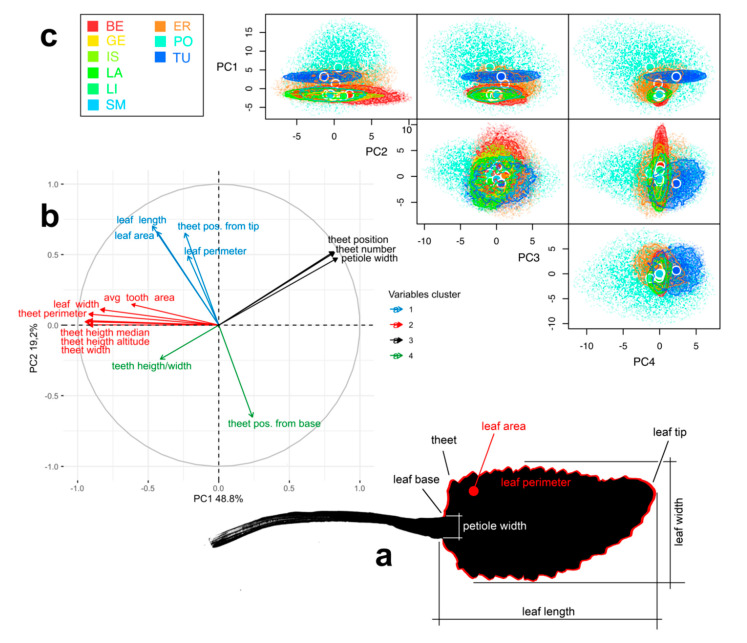
Leaf morphology: (**a**) Sketch of a *Tephroseris balbisiana* leaf and the main traits measured. (**b**) PCA on the 15 leaf morphology traits. Each of them is represented as eigenvector and clusterized with k-means analysis based on correlations. (**c**) Pair plots representing the 4-dimensional hypervolumes for each of the nine populations of *T. balbisiana*. For each hypervolume, 5000 stochastic points are shown.

The output of MorphoLeaf consisted of 1425 measures (Table 2) which we summarized in a PCA that explained 67.99% variance on the first two components (PC1 = 48.76% and PC2 = 19.23%) (Figure 3b). Eigenvectors clustered into four groups. Main contributing variables in PC1 formed the cluster 2 (in red), while main contributors of the PC2 were in the cluster 1 (Figure 3b in blue).

The geometry of the multi-dimensional morphospace was overall similar for six out of nine populations (Figure 3c), all of which occurring in the core distribution area. The most morphologically divergent populations were ER, TU and PO, having a lower morphospace overlap (β Total; Table S2) and higher distance between centroids (Table S3). Volume of the morphospaces and trait dispersion was comparable in all populations (between 2.5 and 6.8) except for the PO population, presenting a much ampler trait dispersion (11.3) and two order of magnitude voluminous morphospace due to greater trait variability among individuals (Figure 3c and Table 2).

### 2.4. Species Distribution Modelling

Analysis of multicollinearity among environmental variables revealed a high degree of correlation among predictors (Figure S3). As a result, we created a subset of five non-collinear variables for constructing the models: soil organic material content, soil pH, precipitation seasonality, solar radiation, and daily temperature range. The three modelling algorithms gave similar outputs, although there were minor discrepancies between the most important variables (Table 3).

All models revealed a positive relationship between habitat suitability and solar radiation, with the species being preferentially found in areas with high irradiation. The habitat suitability for the species was also found to be higher at intermediate values of precipitation seasonality (between 15 and 35 in the coefficient of variation of precipitation). Instead, the contribution of the soil variables in constructing the models was negligible (slightly higher contribution in the generalized boosted regression model; Table 3).

**Figure 4:**
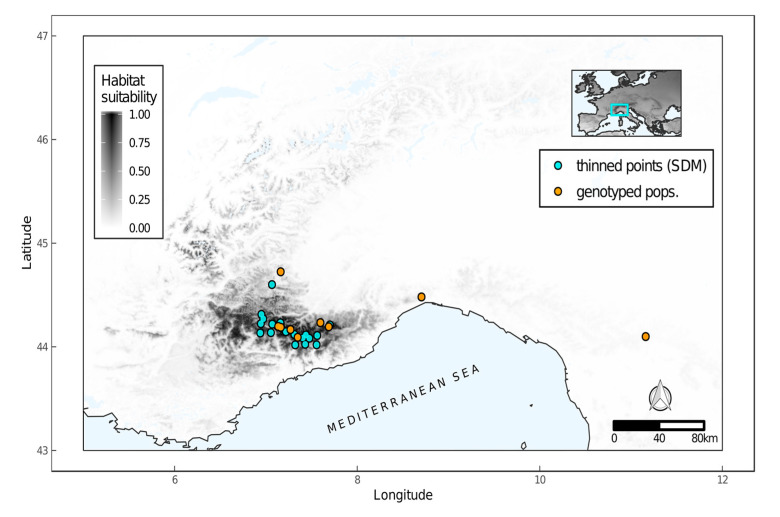
Predicted habitat suitability for *Tephroseris balbisiana* based on ensemble of the results of species distribution models. Gradient of grey gives habitat suitability, with darker areas indicating greater suitability.

All algorithms tested had a good fit, with none clearly outperforming the others in terms of predictive ability (all Boyce indexes > 0.65; mean of the 50 bootstraps). Therefore, we used an ensemble of the three model projections to represent the species distribution. Overall, suitable areas predicted by the model were congruent with the known distribution (Figure 4), with most suitable areas corresponding to the Maritime Alps at the border between Italy and France. The model failed in predicting as suitable the areas in which the species occurs in the Apennines.

## 3. Discussion

According to the Unified Species Concept *sensu* de Queiroz [23,24], populations share a common history and a common future only if a certain level of gene flow is granted. Several factors, however, may hinder this flow. Differential dispersal ability, ecological constraints and historical processes such as geographical isolation and local extinction all may act synergistically in determining the distribution of the different populations across space and time [33] and, ultimately, the connection of their gene pools. The complexity of these events can affect the interpretation of species limits and relationships [34,35] thus frustrating both taxonomic and conservation efforts. Based on these reasons, it is now accepted that to understand variation at the species level in complex taxa, it is better to use a multidisciplinary approach, for example by integrating molecular, ecological and other data [36]. In the present paper, we used molecular fingerprinting based on SCoTs, ITS sequence variation, morphospace analysis and species distribution modelling to investigate the genetic boundaries and geographical patterns of divergence in *T. balbisiana*.

Molecular fingerprinting of the nine populations of *T. balbisiana* led to the separation of six clearly distinct gene pools. This result was partially confirmed by all further survey techniques: ribotyping and morphological analyses revealed higher morphological variability in PO and certain morphological divergence in ER and TU with high cohesion in remaining populations. The SDM analysis, in a similar way, highlighted how Appennine populations has different habitat requirement if compared with Alpine populations.

### 3.1. Genetic Analysis

Genetic structure, obtained by SCoTs fingerprinting, in *T. balbisiana* revealed a coherent group of individuals belonging to the westernmost sector of the Alps, located on the French side of the Argentera-Mercantour massif (Figure 1). This group resulted genetically homogeneous and separated from the Italian populations. *T. balbisiana* in this part of the Alps is continuously distributed and thus, distinctively separated populations are probably rare. Conversely, on the inner side of the Western Alps, most of the populations are isolated, almost probably caused by adaptation to different habitats, and show a greater genetic distance. Furthermore, populations of the inner side of the massif were genetically more differentiated and structured from one another than the previous. Individuals from the Gesso Valley (GE), for example, genetically diverged from the populations of the Ligurian Alps occurring in close proximity (BE and LI) (Figure 1). These populations are commonly associated with permanently humid habitats (including margins of little streams and runoff from melting snow) and they are isolated due to the natural rarity and discontinuity of their habitat. It is possible that differences in environmental requirements (water supply and the chemical nature of the substrates) might have contributed to the differentiation of these populations despite their geographic proximity.

Surprisingly, the PO population of the Cottian Alps, one of the peripheral populations considered in this study, showed the highest genetic diversity. This result was a bit surprising given that genetic diversity is usually lower in isolated populations due to genetic drift, inbreeding, bottlenecks and founder effects [22].

When examining the genetic relation of the ER population of *T. balbisiana* (*sensu* Bonafede et al. [31]) of the Tuscan-Emilian Apennines with *T. longifolia* subsp. *brachychaeta* (syn. *T. italica*) sampled in San Benedetto Val Sambro (BO), our results supported the separation of the two taxa from the rest of the *T. longifolia* aggregate of species (*T. tenuifolia* aggregate *sensu* Pignatti [37]) (Figure S2). With regard to this last finding, our results are congruent with the existing literature on the topic. In fact, *T. longifolia* subsp. *brachychaeta* was found to be the most morphologically and kariologically distinctive species within the *T. longifolia* aggregate taxa [38,39].

Our ITS phylogeny supported the genetic relatedness of the Alpine populations as a single clade sister to the Spanish endemic *T. coincyi* and their divergence from the Apennine populations. Individuals of the TU population (Ligurian Apennines), on the contrary, grouped separately and with statistical support with members of the *T. longifolia* aggregate, thus pointing to the presence of three divergent lineages. The presence of only slightly differences in morphology and high genetic variability is a typical feature of the genus and it is coherent with the literature. Our findings, however, seem to contrast both with the absence of genetic variability found in the plastid genome of *T. balbisiana* and with the morphological identity assessed in the field by local botanists [31]. However, similar chloroplast sharing among closely related species or populations of species also when they possess distinct ploidy levels is not new to the literature (as revised by Naciri and Linder [34]).

As pointed out by Naciri and Linder [34] ancestral polymorphism does exist among closely related species. The phylogenetic affiliations among the Apennine populations and the remaining European taxa shown by this study deserves further and more in-depth analyses also with regard to the role of these mountains during pre- and post-glaciation migrations of taxa in central Europe.

### 3.2. Morphological Analysis

The diverging genetic pattern of peripheral populations of *T. balbisiana* observed in this study found further support as far as we took leaf morphology into consideration. Morphospace characterization demonstrated a strong degree of overlap among Alpine populations, while peripheral populations (PO, TU and ER) were morphologically divergent. While leaf morphology represents a trade-off between the need of maximizing photosynthesis and to minimizing environmental stress, the morphology is also strictly under genetic control [40]. This balance between ecological pressure and evolutionary constraints might result in different levels of diversification among species, populations or even within identical genotypes if subjected to different environmental pressures. We found that morphospaces of TU and ER are highly divergent, not only from the Alpine populations of *T. balbisiana*, but also from one another. In the case of PO from the Cottian Alps, the morphospace expansion testifies a high morphological plasticity among individuals, which is superimposed on the Appenninic and the remaining Alpine populations. Considering that the site in the Cottian Alps encompasses a limited breadth of environmental conditions, we argue that the morphological variability observed for PO would be associated with a higher genetic diversity rather than have a strict ecological origin; a finding that is further corroborated by its isolated genetic status.

A large part of the morphological variance was explained by morphometric parameters linked to leaf margins (teeth number, perimeter and position; Figure S4). Interestingly, leaf margins shape is one of the most important traits to differentiate species in the genus *Tephroseris* [37]. In this context it is possible to identify three different morphospaces; one for the Alpine population (excluding PO), one for the TU population and one for the ER population. The three groups grow in areas with distinct climatic conditions which may explain the variability [41,42,43], but the genetic component most likely has a further influence as emphasized by the genetic results discussed in the previous paragraph.

### 3.3. Species Distribution Modeling

The distribution model was effective in predicting the distribution of the species in its core area on the Alps, whereas Apennine populations were not accounted by the predicted suitable habitat. *Tephroseris balbisiana* core range overlays the distribution of other endemic taxa whose distribution is centered in the Argentera-Mercantour massif [2,5]. Bibliographical data describe *T. balbisiana* habitat as typical of the communities of high altitude megaforbs with flowing water in the soil and a significant amount of organic matter. Our results add another tip to the puzzle of the species habitat description, highlighting the strong influence of the solar radiation.

The second most important variable was precipitation seasonality. Distribution of the precipitation along the year is notably different in Ligurian and Maritime Alps compared to the continental-like precipitation regime found in the rest of the mountain chain [2], being characterized by rainy springs and autumns with a relatively dry summer. This specificity could create a barrier for the species diffusion northward. Both temperature range and soil pH had less influence on the species distribution, as mirrored by the presence of the plant on both acid and basic substrates. Also, the organic matter content of the soil exerted little to null influence on *T. balbisiana* distribution. However, this is an unexpected result because the species is reported in literature to be associated with soils with moderate organic matter content and it is often found in places far from livestock sheltering areas [29,37]. It may be that the spatial resolution of the environmental predictors (approximately 1 km^2^) was not fine enough to capture similar local-scale conditions.

### 3.4. Concluding Remarks

Genetic, morphological and ecological results converged towards identifying similar species boundaries, thus satisfying the Unified Species Concept assumptions.

Specifically, our analysis shows that alpine populations of *T. balbisiana* correspond to a unique species. Apennine populations, on the contrary, are almost probably representative of the genetic and morphological complexity commonly found in the *T. longifolia* and *T. integrifolia* aggregate of species and of their populations which diverged recently and thus had a short time for speciation. Determination of ER individuals as *T. balbisiana* was probably due to a mistake that is not unjustified considering that at least one population from the Alps (PO) shows intermediate characters both at a genetic and morphological level. Moreover, the species distribution modelling shows that this population occurs in an ecologically sub-optimal site. Its variability could be explained by its peripheral position, but we do not exclude a possible hybridization with other *Tephroseris* species, accounting for the similarity with the Apennine populations (Figure 1b).

From a conservation point of view, it is important to highlight that most populations of *T. balbisiana* are concentrated in westernmost Alps, whereas on the inner side of the west Alps they are much more sparse and localized, causing a greater genetic differentiation among populations. However, this genetic uniqueness is endangered by the lack of conservation measures for this species. In two sites investigated the species has not been found. In both sites, water intake structures have dried up what was once the optimal habitat for this species. These findings are symptoms of the mounting anthropic pressure on wet habitats and global freshwater stocks (e.g., Finlayson et al. [44]); in the optic of a sustainable development, we must preserve both freshwater access and species diversity. At present, most of the species’ core distribution area lies within the boundaries of national parks, sites of community importance, and special protection areas, including Parco Naturale Alpi Marittime (Italy), Parco Naturale del Marguareis (Italy) and Parc National du Mercantour (France). Conversely, isolated populations in the Apennines and Cottian alps, which are the most distinctive from a genetic and ecological standpoint, currently lack formal protection. Given that the funding devoted to conservation of these species are limited (if any), we emphasize the importance of redirecting conservation efforts toward these isolated populations.

## 4. Materials and Methods

### 4.1. Sampling Design

During July–August 2019, we sampled between 10 and 30 different individuals of *T. balbisiana* depending on the population size for a total of 75 individuals in 10 sites covering the whole species range (Table 4). We selected the sites according to the following criteria: Three sites in France (locality codes: IS, LA, and SM) where the species is seemingly more widespread and populations of *T. balbisiana* are rarely isolated; the type locality of the species (GE); four populations in the Maritime Alps (AV, BE, LI, and PO) and two in the Appennines (TU, ER). In three additional sites, we could not find individuals probably due to the local extinction of the species.

We sampled plants with three different purposes and following rather different protocols. We stored leaves in silica-gel up to DNA extraction as described by Chase and Hill [45]. For the morphological analysis, we stored basal leaves collected from 95 individuals (Table 4) in a herbarium field-press, ensuring to collect only complete and healthy leaves. We selected only adult leaves, evaluating this parameter basing on two factors: Leaf shape, that it is a conserved and easy-to-recognize trait in adult plants, and the fact that all sampled plants were already flowered and then in their adulthood. We also collected stem leaves in a few sites, but in most cases, they were absent or damaged by animal grazing and thus excluded from the analysis.

### 4.2. DNA Extraction

We performed DNA extraction from 50 mg of dried leaf tissue, by means of the commercial kit NucleoSpin^®^ Plant II by Macherey-Nagel (Düren, Germany) as described in the manufacturer instructions. PL1 lysis buffer was preferred due to the clearer final DNA solution. We adjusted the final concentration of DNA to 30 ng/μL and stored DNA samples at −20 °C.

### 4.3. Genotyping by Fingerprinting

We performed fingerprinting experiment using the SCoT primers developed by Collard and Mackill [46]. Some limitations restrict practical application of SCoTs analysis (e.g., dominance, homology inferences, and artifact fragments) [46], conscious of the method limits we applied the method as described below and we coupled results with ribotyping, morphology, and species distribution modeling, integrating different levels with the goal to obtain a reliable overview of the situation [25].

We selected a total of 5 primers which returned the highest number of bands with the highest composition variability when tested on a subset of samples. We performed Polymerase chain reactions (PCRs) in 25 μL final volumes containing either 1 μL of DNA, 2.5 μL of 10× Taq buffer (Thermo Fisher Scientific, Waltham, MA, USA), 0.1 mmol dNTPs, 2 μmol of primer, 1% bovine serum albumin, 1U of Taq polymerase (Thermo Fisher Scientific), 13.4 μL of water. PCRs were ran in a T3000 Thermocycler by Biometra (Goettingen, Germany) at 94 °C for 3 min, followed by 35 cycles of 1 min at 94 °C, 1 min at 50 °C and 2 min at 72 °C, with a final extension at 72 °C for 5 min. We visualized PCR templates on 1.4% Agar gels stained with ethidium bromide in TAE 0.5× buffer at 80 V for 100 min. We used the peq-GOLD 100 bp plus ladder by VWR (Radnor, USA) as fragment size marker. We visualized gels and acquired images with a Gel Doc™ EZ system by BioRad (Basel, Switzerland). We defined bands size with the help of the software Image Lab Software developed by BioRad and further manually checked by two independent operators. Due to the high number of bands obtained from each marker, five SCoT primers were enough to obtain a significant number of loci to perform genetic population analysis. Fingerprinting first output was a loci presence-absence matrix (Table S4).

Except when otherwise stated, we performed genetic analyses in R version 3.6.2 (R Core Team, 2019) and the package “poppr” [47]. We used Table S4 to perform the following analysis. We calculated a genotype accumulation curves for determining the minimum number of loci necessary to discriminate between individuals in a population, using the *genotype_curve* function with 1000 resamples. We applied the *clonecorrect* function to identify duplicated multilocus genotypes into the original dataset and the *informloci* function to remove phylogenetically uninformative loci. We performed Analysis of Molecular Variance (AMOVA) to estimate molecular variance among and between samples and populations, using the *poppr.amova* function and the *randtest.amova* function to verify the covariance components (1000 permutations). We characterized each population with several indices for heterozygosity, evenness, and linkage with the *poppr* function. Bayesian clustering, implemented in STRUCTURE v2.3 [48], was used to infer the number of putative genetic clusters. We ran the final simulations with 1 < K < 12 and 30 iterations for each K value; each run comprised a burn-in period of 10^5^ iterations, followed by 10^6^ Markov chain Monte Carlo steps. Following Evanno et al. [49], we defined the most adequate value for the number of clusters K with the online tool STRUCTURE HARVESTER [50].

### 4.4. Genotyping by Plastid DNA Markers and ITS-Ribotyping

We studied genetic variations among individuals and populations of *T. balbisiana* with both fingerprinting and nucleotides composition variability on several phylogenetic markers. We amplified one ribosomal marker (ITS) and three plastidial markers (trnL, psbB, and psbC) (Table S5) from three individuals of each population. We performed PCRs in 25 μL final volumes containing either 1 μL of DNA, 2.5 μL of 10× Taq buffer (Thermo Fisher Scientific), 0.1 mmol dNTPs, 2 μmol of forward and reverse primers, 0.3% bovine serum albumin, 1 U of Taq polymerase (Thermo Fisher Scientific), 13.4 μL of water. PCRs were run in a T3000 Thermocycler by Biometra (Goettingen, Germany) at 94 °C for 5 min, followed by 35 cycles of 1 min at 94 °C, 1 min at annealing temperature (Table S5) and 5 min at 72 °C, with a final extension at 72 °C for 10 min. We purified PCRs templates using the NucleoSpin^®^ Gel and PCR Clean-up by Macherey-Nagel following manufacturer instructions. Fragments were sized between 400 and 1400 bp depending of the marker. The purified PCR templates were Sanger sequenced by Bio-Fab Research s.r.l. (Rome, Italy). We analyzed sequence chromatograms with SeqTrace 0.8.1 [51] and retained high quality bases only. Due to the absence of variability in plastidial markers, we will hereafter refer to this analysis as “ribotyping” instead of nucleotide composition variations on ribosomal sequences. We aligned ITS sequences with Muscle [52] and manually inspected variations in nucleotides composition. Each different sequence was identified as a different ribotype.

In order to better interpret ribotypes differences among populations, we analyzed ITS sequences of *T. balbisiana* together with sequences belonging to different congeneric species available on the National Center for Biotechnology Information (NCBI) sequence database. We aligned sequences with Muscle and selected the most appropriate substitution model with JModelTest 2 [53]. We inferred the phylogentic tree with Seaview 4.0 [54] and MrBayes v3.2.7a [55].

### 4.5. Leaf Morphology

We used leaf traits to explore morphological differentiation among populations of *T. balbisiana*. We choose to use leaf traits to approximate species morphology considering their importance in species delimitation of European *Tephroseris* [37]. We used an Epson Perfection 2450 Photo scanner to acquire leaves images with a 300dpi resolution. We automatically analyzed leaves using MorphoLeaf plug-in v. 1.15 [56] of Free-D v. 1.15 [57]. This plug-in automatically measure global leaf traits including leaf margin traits, where most of the differences occurs. We measured 15 leaf traits (Table 2) from a minimum of nine leaves/population (Table 4), each leaf corresponding to a different individual. We used a 40% fidelity ratio to assess leaf contour. We applied sinus extraction with default settings and tips extraction using the local symmetry maximum. Teeth hierarchy was determined by means of the hierarchy iterative method with default settings.

To assess the morphological overlap between the nine populations of *T. balbisiana*, we analyzed variations in leaf morphology in a multi-dimensional morphospace [58,59]. To avoid inter-correlation effects in the morphometric data, we calculated a principal component analysis (PCA) using prcomp function from the R “base” package. We clusterized PCA eigenvectors based on the correlation in four groups by means of a K-means analysis using *kmeans* function from the “base” R package.

We selected the first four principal components (88.5% variance cumulatively explained) to delineate the 4-dimensional morphological hypervolume of each population [59]. We constructed hypervolumes via a Gaussian kernel density estimator, assessing optimal bandwidth value for each axis with the cross-validation method [60]. Following [61], we calculated morphospace differentiation using one overlap and one distance metric, respectively the pairwise overall differentiation among hypervolumes (β total; [62,63] and the distance between centroids. We further calculated trait dispersion for each population with the *kernel.dispersion* function in the “BAT” R package [64] and default settings [63].

### 4.6. Species Distribution Modeling

#### 4.6.1. Occurrence Data

We assembled a dataset of 277 occurrence localities of *T. balbisiana* based on literature data, herbarium collections, and original data [Silene-Flore (http://flore.silene.eu), exported on December 2018]. Occurrences distribution was heterogeneous, with several proximate localities at a distance lower than to the spatial resolution of our environmental rasters, and a disproportion of occurrences in the French side of the distribution range of the species (13 records in Italy vs. 264 in France). This disproportion is partly originated by the different accession data policies in force in the two countries, but to some extent it is also coherent with species distribution. In order to minimize spatial bias, we performed a thinning of the occurrences using the *thin* function in the R package “red” [65], setting the resolution at 0.008 (namely the spatial resolution of environmental predictors). This operation reduced the database to 33 spatially independent occurrences, which we used for modelling the species distribution.

#### 4.6.2. Calibration Area

We calibrated species distribution models within the accessible area [66,67], namely the geographical extent hypothesized to fall within the long-term dispersal and colonization potential for *T. balbisiana* over its evolutionary history. In lack of precise information on the dispersal ability of this species, we used the maximum linear distance between any occurrences in our dataset (ca. 250 km) as a general indication of the maximum dispersal potential. We buffered thinned occurrence records by this distance and combined all individual buffers in a single envelope that we used to mask the environmental predictors. Given that the species does not occur at low altitudes, we further excluded from the accessible area areas below 550 m a.s.l. (Figure 1a).

#### 4.6.3. Environmental Predictors

We used a combination of climatic, topographical and soil variables as predictors (Figure S2), all variables at a resolution of 30 arc-seconds (~1 km^2^ at the equator). To represent topography, we extracted a standard elevation data raster layer from the WorldClim2 [68]. We also extracted climatic variables from WorldClim2 [68], namely Isothermality, Average annual precipitations, Precipitation seasonality, Mean annual temperature, Annual and Daily temperature range, and Water vapor pressure, Solar radiation and Wind speed. We extracted soil variables from the SoilGrids database [69]: Organic soil content and pH, both measured at a 15 cm depth. There variables are at a resolution of 250 m, so we upscaled them to the resolution of climatic variables. We explored collinearity in the initial set of predictors [70] using Pearson correlations, setting the threshold for collinearity at |r| > 0.7 [32]. We excluded collinear variables based on our expert opinion, namely we retained those variable that we considered more representative of species biology and life history traits.

#### 4.6.4. Modelling Procedure

Following Mammola et al. [5,71], we modelled the species distribution using three standard models belonging to the three main categories of algorithms for species distribution modelling: Regression (generalized additive models; [72]), regression trees (generalized boosted regression models; [73]), and machine-learning (maximum entropy model—MaxEnt; Phillips et al. [74]).

We followed the modelling protocol described in [5]. We estimated model performance by running 50 replicates of models, keeping a random partition of 20% of the points for each run to assess the predictive ability. Predictive ability of the three models was obtained by calculating the Boyce index [75]. We projected the model into the accessible area to obtain a graphical representation of the current distribution of *T. balbisiana*. Since model performance was similar among the three techniques, we used an ensemble of the three model projections to represent the species distribution [76,77]—median value of the three individual projections weighted by the Boyce index.

## Figures and Tables

**Table 1:** AMOVA results: the sigma represents the variance, σ, for each population and the percent of the total variance explained by each source of variance. Phi provides the population differentiation statistics. We would expect a higher Phi value to represent a higher amount of differentiation. Sigma and Phi values are statistically significant.

Source of Variation	Sigma	%	Phi	*p* Value
Variations Between Populations	1.132	7.693	0.077	0.040
Variations Between samples within Populations	5.382	36.570	0.396	0.001
Variations within samples	8.203	55.736	0.443	0.001
Total variations	14.718	100.000		

**Table 2:** Characters measured in leaf morphology: The average value (± standard deviation) of each measurement made on the leaves is reported separately for each population. The “avg” in front of the measurement name indicate that the value was used as an average value in the principal component analysis. The unit of measurement is given in square brackets. LA, IS, SM, and GE populations were from Maritime Alps; LI and BE were from Ligurian Alps; PO was from Cottian Alps and TU and ER were from Appennines. See Table 4 for populations details.

Measure	BE	ER	GE	LA	LI	PO	SM	TU	IS
Leaf length (mm)	177.1 ± 36.5	160.4 ± 37.6	121.3 ± 16.7	119.0 ± 27.9	163.6 ± 17.1	1419 ± 24.4	143.9 ± 24.3	118.3 ± 39.9	120.0 ± 31.7
Leaf width (mm)	16.2 ± 5.5	41.9 ± 26.1	13.9 ± 4.9	16.4 ± 4.3	66.9 ± 5.7	62.2 ± 72.4	45.2 ± 3.4	35.5 ± 23.8	14.6 ± 6.0
Leaf area [mm^2^]	883.0 ± 231.4	918.1 ± 245.9	708.1 ± 115.6	656.3 ± 148.8	926.9 ± 100.3	839.3 ± 228.7	716.3 ± 118.4	639.1 ± 186.9	699.9 ± 183.7
Leaf perimeter (mm)	258 ± 92	256 ± 118	174 ± 53	192 ± 105	208 ± 96	205 ± 34	279 ± 69	225 ± 102	174 ± 86
Petiole width (mm)	0.14 ± 0.04	0.07 ± 0.03	0.14 ± 0.02	0.12 ± 0.03	0.09 ± 0.02	0.07 ± 0.02	0.06 ± 0.02	0.09 ± 0.01	0.13 ± 0.02
Teeth number	66 ± 22	34 ± 12	62 ± 9	56 ± 12	45 ± 7	29 ± 7	29 ± 10	40 ± 5	62 ± 10
Avg tooth position (mm)	0.07 ± 0.02	0.04 ± 0.01	0.07 ± 0.01	0.06 ± 0.01	0.05 ± 0.01	0.03 ± 0.01	0.03 ± 0.01	0.05 ± 0.01	0.07 ± 0.01
Avg tooth width (mm)	106 ± 11	155 ± 53	93 ± 8	95 ± 12	252 ± 4	317 ± 269	246 ± 12	202 ± 75	88 ± 10
Avg tooth height altitude (mm)	19 ± 4	59 ± 14	17 ± 4	13 ± 6	37 ± 2	48 ± 34	40 ± 5	30 ± 12	15 ± 3
Avg tooth height median (mm)	28 ± 4	76 ± 24	25 ± 4	21 ± 6	71 ± 2	71 ± 57	55 ± 6	40 ± 13	22 ± 4
Tooth heigth width ratio	0.0007 ± 0.0001	0.0017 ± 0.0003	0.0007 ± 0.0001	0.0006 ± 0.0002	0.0006 ± 0.0001	0.0008 ± 0.0002	0.0185 ± 0.0001	0.0106 ± 0.0085	0.0007 ± 0.0001
Avg tooth area [mm^2^]	30.4 ± 9.9	118.5 ± 72.3	26.1 ± 6.7	16.6 ± 11.2	29.1 ± 3.3	76.3 ± 15.4	11.8 ± 11.0	12.1 ± 3.2	16.1 ± 4.7
Avg tooth perimeter (mm)	12.8 ± 1.4	23.6 ± 6.8	11.4 ± 1.1	11.1 ± 1.8	30.8 ± 0.6	39.5 ± 33.5	29.0 ± 1.7	23.8 ± 7.8	10.6 ± 1.3
Avg tooth position from tip (mm)	0.23 ± 0.01	0.2 ± 0.03	0.22 ± 0.01	0.21 ± 0.01	0.22 ± 0.01	0.22 ± 0.04	0.21 ± 0.01	0.21 ± 0.02	0.22 ± 0.01
Avg tooth position from base (mm)	0.21 ± 0.01	0.23 ± 0.03	0.21 ± 0.01	0.22 ± 0.01	0.21 ± 0.01	0.21 ± 0.04	0.22 ± 0.01	0.22 ± 0.02	0.21 ± 0.01
Morphospace volume	725.68	4597.89	52.79	89.72	294.76	31.16	41056.2	142.77	119.43
Morphospace dispersion	5.54	6.84	3.17	3.92	2.62	11.24	3.39	5.21	4.10

**Table 3:** Set of variables selected for constructing the species distribution models and their relative importance according to the three algorithms considered: Estimated parameters and variable contributions are only given for non collinear variables (all pairwise Pearson |r| < 0.7 [32]) introduced in the models (Figure S2 in Supplementary Material). For the Generalized additive model, estimated β (±s.d.) and *p*-values are given for the linear terms, whereas estimated degrees of freedoms (edf) and *p*-values based on χ^2^ test for the smoothed terms. For the Generalized boosted regression mode and the maximum entropy model MaxEnt, the variable relative influence and permutation importance are reported, respectively.

Variable	Generalized Additive Model	Generalized Boosted Regression Model	MaxEnt
Precipitation seasonality	edf = 5.26; χ^2^ = 45.46; *p* < 0.001	20.84	15.9
Daily temperature range	edf = 2.48; χ^2^ = 9.27; *p* = 0.03	9.44	7.9
Solar Radiation	0.02 ± 0.04; *p* < 0.001	48.43	73.8
Organic material content	–0.03 ± 0.02; *p* = 0.11	7.93	0.5
pH	–0.11 ± 0.08; *p* = 0.15	13.34	1.9

**Table 4:** Populations description: samples are from ten different populations. For each population are reported (from left to right column): the populations ID; site names; location details; sampling dates; approximate number of individuals; number of samples (individuals) genotyped with start codon targeted polymorphism (SCoT); number of leaves (individuals) measured for the leaf morphology analysis; number of samples (individuals) genotyped with single-nucleotide polymorphisms research in the nuclear ribosomal internal transcribed spacer marker sequence.

Pop ID	Site	Locality, Province, Country	Date	Number of Individuals	SCoTs Genotyping	Leaf Morphology	Ribotyping
AV	Lago Aver Superiore	Vinadio, CN, IT	17 July	-	-	-	1
BE	Vallone S. Bernardo	Limone P.te, CN, IT	19 June	30	10	9	3
ER	Fontana Bura	San Benedetto Val Sambro, BO, IT	19 June	100	10	19	3
GE	Pian della Casa	Valdieri, CN, IT	19 July	12	10	12	3
IS	Isola 2000	Isola, 06, FR	19 September	15	8	11	3
LA	La Lausetta	Isola, 06, FR	19 September	15	6	9	3
LI	Porta Sestrera	Chiusa di Pesio, CN, IT	19 July	50	10	8	3
PO	Rio Ratisin	Crissolo, CN, IT	19 July	100	12	9	3
SM	La Pointe	Saint-Martin Vesubie, 06, FR	19 September	15	4	7	3
TU	Passo del Turchino	Masone, GE, IT	19 July	20	5	11	3

## Supplementary Materials

The following are available online at https://www.mdpi.com/2223-7747/9/9/1160/s1, Table S1: Genetic diversity indexes, Table S2: Morphological overlap, Table S3: Morphological distance, Table S4: GenAlex formatted matrix for fingerprinting analysis, Table S5: Primers details, Figure S1: Genotype Accumulation Curve, Figure S2: ITS based phylogeny of Tephroseris genus, Figure S3: Collinearity among predictors, Figure S4: Contribution of variables to PCs.
