# Supplementary material for: Integrating Multiple Lines of Evidence to Explore Intraspecific Variability in a Rare Endemic Alpine Plant and Implications for Its Conservation
Source: Plants (Basel). 2020 Sep 8;9(9):1160. doi: 10.3390/plants9091160 (PMC7569986; doi:10.3390/plants9091160)

SUPPLEMENTARY MATERIALS

SUPPLEMENTARY TABLES

Table S1: Genetic diversity indexes

Table S2: Morphological overlap

Table S3: Morphological distance

Table S4: GenAlex formatted matrix for fingerprinting analysis

Table S5: Primers details

SUPPLEMENTARY FIGURES

Figure S1: Genotype Accumulation Curve

Figure S2: ITS based phylogeny of *Tephroseris* genus

Figure S3: Collinearity among predictors

Figure S4: Contribution of variables to PCs

SUPPLEMENTARY TABLES

Table S1. Genetic diversity indexes

Pop	MLG	H	G	lambda	E.5	Hexp	Ia	rbarD
BE	10	2.3	10	0.9	1	0.123	1.568	0.0832
GE	9	2.16	8.33	0.88	0.952	0.111	1.057	0.0713
LI	10	2.3	10	0.9	1	0.247	2.019	0.0654
PO	12	2.48	12	0.917	1	0.233	1.958	0.068
TU	5	1.61	5	0.8	1	0.203	4.41	0.1764
ER	9	2.16	8.33	0.88	0.952	0.249	4.004	0.134
SM	4	1.39	4	0.75	1	0.195	0.265	0.0126
LA	6	1.79	6	0.833	1	0.155	0.272	0.0151
IS	8	2.08	8	0.875	1	0.142	1.745	0.1027
Total	73	4.28	71.2	0.986	0.985	0.332	2.261	0.0411

Pop Population name.

MLG Number of multilocus genotypes (MLG) observed.

H Shannon-Wiener Index of MLG diversity (Shannon, 1948).

G Stoddart and Taylor's Index of MLG diversity (Stoddart & Taylor, 1988).

lambda Simpson's Index (Simpson, 1949).

E.5 Evenness, E5 (Pielou, 1975; Ludwig & Reynolds, 1988; Grünwald et al., 2003).

Hexp Nei's unbiased gene diversity (Nei, 1978).

Ia The index of association, IA (Brown, Feldman & Nevo, 1980; Smith et al., 1993).

rbarD The standardized index of association, r¯d[@].

References

Grünwald NJ., Goodwin SB., Milgroom MG., Fry WE. 2003. Analysis of genotypic diversity data for populations of microorganisms. Phytopathology 93:738–746.

Ludwig JA., Reynolds JF. 1988. Statistical ecology: A primer in methods and computing. Wiley.com.

Nei M. 1978. Estimation of average heterozygosity and genetic distance from a small number of individuals. Genetics 89:583–590.

Pielou EC. 1975. Ecological diversity. Wiley New York.

Shannon CE. 2001. A mathematical theory of communication. ACM SIGMOBILE Mobile Computing and Communications Review 5:3–55.

Simpson EH. 1949. Measurement of diversity. Nature 163:688.

Smith JM., Smith NH., O'Rourke M., Spratt BG. 1993. How clonal are bacteria. Proceedings of the National Academy of Sciences 90:4384–4388.

Stoddart JA., Taylor JF. 1988. Genotypic diversity: Estimation and prediction in samples. Genetics 118:705–711.

Table S2. Morphological overlap: Pairwise β total (Carvahlo & Cardoso, 2020; Mammola & Cardoso, 2020) values among the morphospaces of the nine populations

	**BE**	**ER**	**GE**	**IS**	**LA**	**LI**	**PO**	**SM**	**TU**
**BE**	0.00	0.89	0.92	0.88	0.68	0.96	0.98	0.81	1.00
**ER**	0.89	0.00	0.99	0.98	0.94	0.99	0.89	0.97	0.87
**GE**	0.93	0.98	0.00	0.57	0.84	0.61	0.99	0.70	1.00
**IS**	0.88	0.98	0.57	0.00	0.75	0.69	0.99	0.62	1.00
**LA**	0.67	0.94	0.84	0.75	0.00	0.90	0.99	0.59	1.00
**LI**	0.95	0.99	0.61	0.69	0.90	0.00	0.99	0.81	1.00
**PO**	0.98	0.89	0.99	0.99	0.99	0.99	0.00	0.99	0.97
**SM**	0.80	0.96	0.69	0.62	0.59	0.81	0.99	0.00	1.00
**TU**	1.00	0.87	1.00	1.00	1.00	1.00	0.97	1.00	0.00

Table S3. Morphological distance: Pairwise distances between centroids among the morphospaces of the nine populations.

	BE	ER	GE	IS	LA	LI	PO	SM	TU
**BE**	0.0	3.60	1.92	2.28	3.12	0.95	7.55	2.64	6.25
**ER**	3.60	0.00	3.93	3.65	3.71	3.74	4.88	3.20	3.49
**GE**	1.92	3.93	0.00	0.67	1.47	0.98	7.83	1.35	6.25
**IS**	2.28	3.65	0.67	0.00	0.99	1.42	7.72	0.72	5.88
**LA**	3.12	3.71	1.47	0.99	0.00	2.29	7.45	0.82	5.50
**LI**	0.95	3.74	0.98	1.42	2.29	0.00	7.74	1.92	6.25
**PO**	7.55	4.88	7.83	7.72	7.45	7.74	0.00	7.36	5.00
**SM**	2.64	3.20	1.35	0.72	0.82	1.92	7.36	0.00	5.29
**TU**	6.25	3.49	6.25	5.88	5.50	6.25	5.00	5.29	0.00

Table S4. GenAlex formatted matrix used for fingerprinting analysis (csv format)

66,75,9,10,10,10,12,5,10,4,6,8,,

,,,BE,GE,LI,PO,TU,ER,SM,LA,IS,,

Ind,Pop,11_1,11_2,11_3,11_4,11_5,11_6,11_7,11_8,11_9,11_10,11_11,11_12,11_13,11_14,11_15,11_16,11_17,11_18,11_19,11_20,11_21,11_22,11_23,11_24,11_25,12_1,12_2,12_3,12_4,12_5,12_6,12_7,12_8,12_9,12_10,12_11,12_12,14_01,14_02,14_03,14_04,14_05,14_06,14_07,14_08,14_09,14_10,14_11,14_12,14_13,14_14,14_15,14_16,14_17,14_18,14_19,14_20,14_21,15_1,15_2,15_3,15_4,15_5,15_6,15_7,15_8

BE01,BE,0,0,1,0,1,0,0,1,0,1,1,1,1,1,1,1,0,1,0,0,1,0,1,1,0,0,0,0,0,1,0,0,0,0,0,1,1,1,0,1,0,0,1,0,1,1,0,0,0,0,0,1,0,0,0,1,1,0,0,1,0,1,1

BE02,BE,0,0,0,1,1,1,0,1,0,1,1,1,1,0,1,1,1,0,1,0,0,1,0,1,1,0,1,0,0,1,0,1,1,0,0,0,0,1,1,1,0,1,0,0,1,0,1,1,0,1,0,0,1,0,1,1,0,0,1,1,0,0,1,1,1,1

BE03,BE,1,0,0,1,0,1,0,1,0,1,1,1,1,0,1,1,1,0,1,0,0,1,0,1,1,0,1,0,0,1,0,1,1,0,0,0,0,1,1,1,0,1,0,0,1,0,1,1,0,1,0,0,1,0,1,1,0,0,1,1,0,0,1,0,1,1

BE04,BE,0,0,1,0,1,1,0,1,0,1,1,1,1,0,1,1,1,0,1,0,1,1,0,1,1,1,1,0,0,1,0,1,1,0,0,0,0,1,1,1,0,1,0,1,1,0,1,1,1,1,0,0,1,0,1,1,0,0,1,1,0,0,1,0,1,1

BE05,BE,0,0,0,1,0,1,0,1,0,1,1,1,1,1,1,1,1,0,1,0,0,1,1,1,1,0,1,0,0,1,1,1,1,0,0,0,0,1,1,1,0,1,0,0,1,1,1,1,0,1,0,0,1,1,1,1,0,0,1,1,0,0,1,1,1,1

BE06,BE,0,0,1,0,1,0,0,1,0,1,1,1,1,1,1,1,1,0,1,0,0,1,1,1,1,0,1,0,0,1,1,1,1,0,0,0,0,1,1,1,0,1,0,0,1,1,1,1,0,1,0,0,1,1,1,1,0,0,1,1,0,0,1,0,1,1

BE07,BE,0,0,0,1,1,1,0,1,0,1,1,1,1,0,1,1,1,0,1,0,0,1,0,1,1,0,0,0,0,1,0,1,1,0,0,0,0,1,1,1,0,1,0,0,1,0,1,1,0,0,0,0,1,0,1,1,0,0,1,1,0,0,1,0,1,1

BE08,BE,1,0,1,0,0,1,0,1,0,1,1,1,1,1,1,1,1,0,1,0,0,1,0,1,1,0,0,0,0,1,0,1,1,0,0,0,0,1,1,1,0,1,0,0,1,0,1,1,0,0,0,0,1,0,1,1,0,0,1,1,0,0,1,1,1,1

BE09,BE,0,0,0,1,0,1,0,1,0,1,1,1,1,0,1,1,1,0,1,0,0,1,0,1,1,1,1,0,0,1,0,1,1,0,0,0,0,1,1,1,0,1,0,0,1,0,1,1,1,1,0,0,1,0,1,1,0,0,1,1,0,0,1,1,1,1

BE10,BE,0,0,0,1,0,1,0,1,0,1,1,1,1,0,1,1,1,0,1,0,0,1,0,1,1,0,1,0,0,1,0,1,1,0,0,0,0,1,1,1,0,1,0,0,1,0,1,1,0,1,0,0,1,0,1,1,0,0,1,1,0,0,1,1,1,1

GE01,GE,0,1,0,0,0,1,0,0,1,0,0,0,0,1,0,0,0,1,0,0,0,0,0,0,0,0,1,0,0,0,0,1,0,0,0,0,0,0,0,0,1,0,0,0,0,0,0,0,0,1,0,0,0,0,1,0,0,0,0,0,0,0,0,0,1,1

GE02,GE,0,1,0,0,0,1,0,0,1,0,0,0,0,1,0,1,0,1,0,0,0,0,0,0,0,1,1,1,0,1,0,1,0,0,0,0,0,0,0,1,0,1,0,0,0,0,0,0,0,1,1,1,0,1,0,1,0,0,0,0,0,0,0,0,0,1,1,1

GE03,GE,0,1,0,0,0,1,0,0,1,0,0,0,0,1,0,1,0,1,0,0,0,0,0,0,0,0,0,0,0,0,1,0,1,0,0,0,0,0,0,0,1,0,1,0,0,0,0,0,0,0,0,0,0,0,0,0,1,0,1,0,0,0,0,0,0,0,0,0,0,0,1,1

GE04,GE,0,1,0,0,0,1,0,0,1,0,0,0,0,1,0,0,0,1,0,0,0,0,0,0,0,1,1,1,0,1,0,1,0,0,1,0,0,0,0,0,1,0,0,0,0,0,0,0,1,1,1,0,1,0,1,0,0,1,0,0,0,1,1,1,1,1

GE05,GE,0,1,0,0,0,1,0,0,1,0,0,0,1,1,0,0,0,1,0,0,0,0,0,0,0,1,1,1,0,1,0,1,0,0,0,0,0,0,0,0,0,1,0,0,0,0,0,0,0,1,1,1,0,1,0,1,0,0,0,0,0,0,1,1,0,1,1

GE06,GE,0,1,0,0,0,1,0,0,1,0,0,0,1,1,0,1,0,1,0,0,0,0,0,0,0,0,0,0,0,0,1,0,1,0,0,0,0,0,0,0,1,0,1,0,0,0,0,0,0,0,0,0,0,0,0,0,1,0,1,0,0,0,0,0,0,0,0,0,0,0,1,1

GE07,GE,0,0,0,0,0,0,0,0,0,0,0,0,0,1,1,0,0,0,1,0,0,0,0,0,0,0,0,0,0,0,0,0,1,0,1,0,0,0,0,0,0,0,0,0,0,1,0,0,0,0,0,0,0,0,0,0,0,0,0,1,0,1,0,0,0,0,0,0,0,0,0,0,0,1,1

GE08,GE,0,0,0,0,0,1,0,0,1,0,0,0,0,1,0,1,0,1,0,0,0,0,0,0,0,1,0,1,0,1,0,1,0,0,0,0,0,0,0,1,0,1,0,0,0,0,0,0,0,1,0,1,0,1,0,1,0,0,0,1,0,0,0,0,0,1,1

GE09,GE,0,0,0,0,0,1,0,0,1,0,0,0,0,1,0,1,0,1,0,0,0,0,0,0,0,1,1,0,1,0,1,0,0,0,0,0,0,1,0,1,0,0,0,0,0,0,0,1,1,0,1,0,1,0,0,0,0,0,0,0,0,0,0,1,1

GE10,GE,0,0,0,0,0,1,0,0,1,0,0,0,0,1,0,1,0,1,0,0,0,0,0,0,0,1,1,0,1,0,1,0,0,0,0,0,0,1,0,1,0,0,0,0,0,0,0,1,1,0,1,0,1,0,0,0,0,0,0,0,0,0,0,1,1

LI01,LI,0,0,0,0,0,0,1,0,0,1,0,0,0,1,1,1,0,0,1,0,0,0,1,0,1,0,0,1,0,1,0,1,0,0,0,0,0,1,1,0,0,1,0,0,0,1,0,1,0,0,1,0,1,0,1,0,0,0,0,0,0,0,1,1,1,1

LI02,LI,0,0,0,0,0,0,1,0,1,1,0,0,0,1,1,1,0,0,1,1,0,0,1,0,1,1,0,0,0,1,0,1,0,0,0,0,0,1,1,0,0,1,1,0,0,1,0,1,1,0,0,0,1,0,1,0,0,0,1,0,0,0,0,1,1,1

LI03,LI,0,0,0,0,0,0,1,0,0,0,0,0,0,1,1,1,1,0,0,0,0,0,0,0,0,1,1,1,0,1,0,1,0,0,0,0,0,1,1,1,0,0,0,0,0,0,0,0,1,1,1,0,1,0,1,0,0,0,1,0,0,0,0,1,1,1

LI04,LI,0,0,0,0,0,0,1,0,1,0,0,0,0,1,1,1,0,0,1,0,1,0,1,0,1,0,0,0,0,1,0,1,1,0,0,0,0,1,1,0,0,1,0,1,0,1,0,1,0,0,0,0,1,0,1,1,0,0,1,0,0,1,0,1,1,1

LI05,LI,0,0,0,0,0,1,0,0,0,0,0,0,0,1,1,1,0,1,0,1,1,0,1,0,1,0,0,0,0,1,1,1,1,0,0,0,0,1,1,0,1,0,1,1,0,1,0,1,0,0,0,0,1,1,1,1,0,0,0,0,0,0,1,1,1

LI06,LI,1,0,1,0,0,1,1,0,0,1,0,0,0,1,1,1,1,1,0,0,0,0,0,1,1,1,1,0,1,1,1,1,0,0,0,0,1,1,1,1,0,0,0,0,0,1,1,1,1,0,1,1,1,1,0,0,0,0,0,0,1,1,1

LI07,LI,0,0,0,0,0,1,0,0,0,1,0,0,0,1,0,1,1,0,1,0,1,0,1,0,1,1,1,0,0,1,0,1,1,0,0,0,0,0,1,1,0,1,0,1,0,1,0,1,1,1,0,0,1,0,1,1,0,0,1,0,0,1,1,1,1,1

LI08,LI,0,0,0,0,0,0,0,0,0,0,0,0,0,1,1,1,1,1,0,0,0,0,0,0,0,0,0,1,0,1,0,1,1,0,0,0,0,1,1,1,1,0,0,0,0,0,0,0,0,0,1,0,1,0,1,1,0,0,0,0,0,0,1,1,1

LI09,LI,0,0,0,0,0,1,0,0,1,0,0,0,0,1,1,1,0,0,1,0,0,0,1,0,1,0,0,1,0,1,0,1,1,0,0,0,0,1,1,0,0,1,0,0,0,1,0,1,0,0,1,0,1,0,1,1,0,0,0,0,0,1,1,1,1

LI10,LI,0,0,0,0,0,1,0,0,0,0,0,0,0,1,1,1,0,1,0,0,1,0,1,0,1,0,0,0,0,1,0,1,1,0,0,0,0,1,1,0,1,0,0,1,0,1,0,1,0,0,0,0,1,0,1,1,0,0,1,0,0,0,1,1,1,1

PO01,PO,0,0,0,0,0,0,0,0,0,0,1,0,0,1,0,0,0,0,1,1,0,0,0,0,1,0,1,0,0,1,0,1,1,0,0,0,0,0,0,0,0,1,1,0,0,0,0,1,0,1,0,0,1,0,1,1,0,0,1,0,0,1,1,0,0,0

PO02,PO,0,0,0,0,0,0,0,0,0,0,1,0,0,1,0,0,0,0,1,1,0,0,0,0,1,0,0,0,0,1,0,1,1,0,0,0,0,0,0,0,0,1,1,0,0,0,0,1,0,0,0,0,1,0,1,1,0,0,0,0,0,1,1,1,1,0

PO03,PO,0,0,0,0,0,0,0,0,0,0,0,0,0,1,0,0,0,0,1,0,0,1,0,0,0,1,1,1,0,1,1,1,0,0,1,0,0,0,0,0,0,1,0,0,1,0,0,0,1,1,1,0,1,1,1,0,0,1,1,0,0,1,1,0,0,0

PO04,PO,0,0,0,0,0,0,0,0,0,0,0,0,0,1,0,0,0,1,1,0,0,0,0,0,0,1,1,0,0,1,0,1,1,0,0,0,0,0,0,0,1,1,0,0,0,0,0,0,1,1,0,0,1,0,1,1,0,0,1,0,0,1,1,0,0,0

PO05,PO,0,0,0,0,0,0,0,0,0,0,0,0,0,1,0,0,0,1,0,1,0,0,0,0,0,1,1,1,0,1,0,1,1,0,1,0,0,0,0,0,1,0,1,0,0,0,0,0,1,1,1,0,1,0,1,1,0,1,1,0,0,1,1,0,0,0

PO06,PO,1,0,0,0,0,0,0,0,0,0,0,0,0,1,0,0,0,0,1,1,0,1,0,0,0,1,0,0,0,0,0,1,1,0,1,0,0,0,0,0,0,1,1,0,1,0,0,0,1,0,0,0,0,0,1,1,0,1,1,0,0,1,1,0,0,0

PO07,PO,1,0,0,0,0,0,0,0,0,0,0,0,0,0,0,0,0,1,0,1,0,0,0,0,0,1,1,1,0,1,1,1,1,0,1,0,0,0,0,0,1,0,1,0,0,0,0,0,1,1,1,0,1,1,1,1,0,1,1,0,0,1,1,0,0,0

PO08,PO,1,0,0,0,0,0,0,0,0,0,0,0,0,1,0,0,0,1,0,0,0,0,0,0,0,1,0,0,0,1,1,1,1,0,1,0,0,0,0,0,1,0,0,0,0,0,0,0,1,0,0,0,1,1,1,1,0,1,0,0,0,1,1,1,1,0

PO09,PO,1,0,0,0,0,0,0,0,0,0,0,0,0,1,0,1,0,1,0,0,0,0,0,0,0,0,1,1,0,1,1,1,1,0,1,0,0,0,1,0,1,0,0,0,0,0,0,0,0,1,1,0,1,1,1,1,0,1,0,0,0,1,1,1,1,0

PO10,PO,1,0,0,0,0,0,0,0,0,0,1,1,0,1,0,1,0,1,0,0,0,0,0,0,0,0,0,0,0,0,1,1,1,1,0,0,0,0,0,1,0,1,0,0,0,0,0,0,0,0,0,0,0,0,1,1,1,1,0,0,0,0,0,1,1,1,1,0

PO11,PO,1,0,0,0,0,0,0,0,0,0,0,1,0,1,0,0,0,0,1,1,0,1,0,0,1,0,1,1,0,1,1,1,0,0,0,0,0,0,0,0,0,0,1,1,0,1,0,0,1,0,1,1,0,1,1,1,0,0,0,1,0,0,1,1,1,1,0

PO12,PO,1,0,0,0,0,0,0,0,0,0,0,0,0,1,0,0,0,0,1,1,0,1,0,0,1,0,0,0,0,1,0,1,1,0,0,0,0,0,0,0,0,0,1,1,0,1,0,0,1,0,0,0,0,1,0,1,1,0,0,1,0,0,1,1,0,1,1

TU01,TU,1,0,0,0,0,0,0,0,0,0,0,0,0,1,0,1,0,1,1,1,0,1,0,0,1,0,0,1,0,1,1,1,1,0,0,0,1,0,1,0,1,1,1,0,1,0,0,1,0,0,1,0,1,1,1,1,0,0,0,0,0,0,0,0,0,1

TU02,TU,1,0,0,0,0,0,0,0,0,0,1,1,0,1,1,0,0,0,1,0,0,0,1,0,1,0,0,1,0,1,1,1,1,0,0,0,0,1,0,0,0,1,0,0,0,1,0,1,0,0,1,0,1,1,1,1,0,0,0,0,0,0,0,0,0,1

TU03,TU,1,0,0,0,0,0,0,0,0,0,0,1,0,1,1,0,0,0,1,0,0,0,0,0,1,0,1,1,0,1,1,1,1,0,0,0,0,1,0,0,0,1,0,0,0,0,0,1,0,1,1,0,1,1,1,1,0,0,0,0,0,0,0,0,0,1

TU04,TU,1,0,0,0,0,0,0,0,0,0,0,1,0,0,1,0,0,0,1,0,0,0,1,0,1,0,1,1,0,1,0,1,1,0,0,0,0,1,0,0,0,1,0,0,0,1,0,1,0,1,1,0,1,0,1,1,0,0,0,0,0,0,0,0,0,1

TU05,TU,1,0,0,0,0,0,0,0,0,0,0,1,0,0,1,0,0,0,1,0,0,0,1,0,1,0,0,0,0,1,0,0,0,0,0,0,0,1,0,0,0,1,0,0,0,1,0,1,0,0,0,0,1,0,0,0,0,0,0,0,0,0,0,0,0,1

ER01,ER,1,0,0,0,0,0,0,0,0,0,1,0,0,1,1,0,0,0,1,0,0,0,0,0,0,0,0,0,0,0,1,0,0,0,0,0,0,0,1,0,0,0,1,0,0,0,0,0,0,0,0,0,0,0,1,0,0,0,0,0,0,0,0,0,0,0,1

ER02,ER,1,0,0,0,0,0,0,0,1,0,1,0,0,0,1,0,0,0,1,0,0,0,0,0,0,0,0,0,0,0,1,0,0,0,0,0,0,0,1,0,0,0,1,0,0,0,0,0,0,0,0,0,0,0,1,0,0,0,0,0,0,0,0,0,0,0,1

ER03,ER,1,0,0,0,0,0,0,0,1,0,1,0,0,0,1,0,0,0,1,0,0,0,0,0,0,0,0,0,0,0,1,0,0,0,0,0,0,0,1,0,0,0,1,0,0,0,0,0,0,0,0,0,0,0,1,0,0,0,0,0,1,0,0,0,0,0,0,1

ER04,ER,1,0,0,0,0,0,0,0,0,0,1,1,0,1,1,0,0,0,0,1,0,1,0,0,0,0,1,1,0,0,1,1,1,1,0,1,1,1,0,0,0,0,1,0,1,0,0,0,0,1,1,0,0,1,1,1,1,0,1,0,0,0,0,0,0,0,0,1

ER05,ER,1,0,0,0,0,0,0,0,0,0,1,1,0,1,1,0,0,0,0,1,0,1,0,0,0,0,0,0,0,0,0,1,0,0,0,0,0,0,0,0,0,0,0,0,0,1,0,1,0,0,0,0,0,0,0,0,0,1,0,0,0,0,0,0,0,0,0,0,0,0,1

ER06,ER,1,0,0,0,0,0,0,0,0,0,1,0,0,1,1,1,0,0,0,1,0,1,0,0,0,1,0,1,0,0,1,0,0,1,0,0,1,0,1,0,0,0,1,0,1,0,0,0,1,0,1,0,0,1,0,0,1,0,0,0,0,0,0,0,0,0,1

ER07,ER,1,0,0,0,0,0,0,0,0,0,1,1,0,1,0,1,0,0,0,1,0,1,0,0,0,1,1,1,1,0,1,0,1,1,0,0,1,0,1,0,0,0,1,0,1,0,0,0,1,1,1,1,0,1,0,1,1,0,0,0,0,0,0,0,0,0,0,1

ER08,ER,1,0,0,0,0,0,0,0,0,0,0,0,0,1,1,0,0,0,0,1,1,1,0,0,0,1,0,1,1,0,1,1,1,1,0,1,1,0,0,0,0,0,1,1,1,0,0,0,1,0,1,1,0,1,1,1,1,0,1,0,0,0,0,0,0,0,0,1

ER09,ER,1,0,0,0,0,0,0,0,0,0,0,0,1,1,0,0,0,0,1,1,1,0,0,0,1,0,1,0,0,1,1,1,1,0,1,0,0,0,0,0,0,1,1,1,0,0,0,1,0,1,0,0,1,1,1,1,0,1,0,0,0,0,0,0,0,1

ER10,ER,1,0,0,0,0,0,0,0,1,0,0,0,0,1,0,0,0,0,1,1,0,0,0,1,1,0,1,0,0,1,0,1,1,0,0,0,0,0,0,0,0,1,1,0,0,0,1,1,0,1,0,0,1,0,1,1,0,0,0,0,0,0,0,0,0,1

SM1,SM,0,0,0,0,0,1,0,0,0,1,0,0,1,1,0,0,1,0,0,0,0,1,0,0,0,0,0,1,0,1,1,1,1,0,0,0,0,0,0,1,0,0,0,0,1,0,0,0,0,0,1,0,1,1,1,1,0,0,1,0,0,0,1,1,1,1

SM2,SM,0,0,0,0,0,0,0,0,0,0,0,0,0,1,0,0,1,0,1,0,0,1,0,0,1,0,1,1,0,0,1,1,1,0,0,0,0,0,0,1,0,1,0,0,1,0,0,1,0,1,1,0,0,1,1,1,0,0,1,0,0,0,1,1,1,1

SM3,SM,0,0,0,0,0,0,0,0,0,0,0,0,0,1,0,0,1,0,1,0,0,0,0,0,0,0,0,1,0,1,1,1,1,1,1,1,1,0,0,1,0,1,0,0,0,0,0,0,0,0,1,0,1,1,1,1,1,1,1,0,0,0,1,1,1,1

SM4,SM,0,0,0,0,0,0,0,0,0,0,1,0,0,1,0,0,1,0,0,0,0,1,0,0,0,0,0,1,0,1,0,1,1,0,0,0,0,0,0,1,0,0,0,0,1,0,0,0,0,0,1,0,1,0,1,1,0,0,1,0,0,0,1,1,1,1

LA1,LA,0,0,0,0,0,0,0,0,0,1,0,0,0,1,0,0,1,0,0,0,0,0,0,0,1,0,0,1,0,1,0,1,1,0,0,0,0,0,0,1,0,0,0,0,0,0,0,1,0,0,1,0,1,0,1,1,0,0,0,0,0,0,0,0,1,0,1

LA2,LA,0,0,0,0,0,0,0,0,0,0,0,0,0,1,0,0,1,0,0,0,0,0,0,0,0,0,0,0,1,0,1,0,1,1,0,1,1,0,0,0,1,0,0,0,0,0,0,0,0,0,0,0,1,0,1,0,1,1,0,1,0,0,0,0,0,0,1,1,1

LA3,LA,0,0,0,0,0,0,0,0,0,0,0,1,0,1,0,0,1,0,0,0,0,1,0,0,1,0,0,1,1,1,1,1,0,0,0,1,0,0,0,1,0,0,0,0,1,0,0,1,0,0,1,1,1,1,1,0,0,0,0,0,0,0,0,0,1,1,0

LA4,LA,0,0,0,0,0,0,0,0,0,0,0,0,0,0,1,0,0,1,0,1,0,0,0,0,0,0,0,0,1,1,1,1,1,1,0,0,1,0,0,0,1,0,1,0,0,0,0,0,0,0,0,0,1,1,1,1,1,1,0,0,0,0,0,1,0,0,1,1,1

LA5,LA,0,0,0,0,0,0,0,0,0,0,0,0,0,0,1,0,0,1,0,0,0,0,0,0,0,0,0,0,1,1,1,1,1,1,1,0,0,1,0,0,0,1,0,0,0,0,0,0,0,0,0,0,1,1,1,1,1,1,1,0,0,0,0,0,0,0,0,0,1,0,1

LA6,LA,0,0,0,0,0,0,0,0,0,1,0,0,0,1,0,0,1,0,0,0,0,1,0,0,1,0,1,1,1,1,0,1,1,0,0,0,0,0,0,1,0,0,0,0,1,0,0,1,0,1,1,1,1,0,1,1,0,0,0,0,0,0,0,0,1,0,1

IS1,IS,0,0,0,0,0,0,0,0,0,0,0,1,0,1,0,0,1,0,0,0,0,0,0,0,0,0,0,0,1,1,1,1,1,0,0,0,0,0,0,1,0,0,0,0,0,0,0,0,0,0,0,0,1,1,1,1,1,0,0,0,0,0,0,0,0,1,0,1

IS2,IS,0,0,0,0,0,0,0,0,0,0,0,0,1,1,0,0,1,0,0,0,0,0,0,0,0,0,0,0,0,0,1,1,1,0,0,1,0,0,0,0,1,0,0,0,0,0,0,0,0,0,0,0,0,0,0,1,1,1,0,0,1,0,0,0,0,0,0,1,0,1

IS3,IS,0,0,0,0,0,0,0,0,0,0,0,0,0,1,1,0,1,0,1,0,0,1,0,0,0,0,0,0,0,0,1,1,1,0,0,0,0,0,1,0,1,0,1,0,0,1,0,0,0,0,0,0,0,0,1,1,1,0,0,0,0,0,1,0,0,1,0,1

IS4,IS,0,0,0,0,0,0,0,0,0,0,0,0,0,1,1,1,0,1,0,0,0,0,0,0,0,0,0,1,1,0,0,1,1,1,1,0,0,0,0,1,0,1,0,0,0,0,0,0,0,0,0,1,1,0,0,1,1,1,1,0,0,1,0,0,0,0,0,1,0,1

IS5,IS,0,0,0,0,0,0,0,0,0,0,0,0,1,1,0,0,1,0,0,0,0,0,0,0,0,0,0,0,0,0,1,1,1,1,1,0,0,0,0,0,0,1,0,0,0,0,0,0,0,0,0,0,0,0,0,1,1,1,1,1,0,0,1,0,0,0,0,0,1,0,1

IS6,IS,0,0,0,0,0,0,0,0,0,0,0,0,0,1,1,0,1,0,1,0,0,1,0,0,0,0,0,0,0,0,1,1,1,0,0,1,0,0,1,0,1,0,1,0,0,1,0,0,0,0,0,0,0,0,1,1,1,0,0,1,1,0,0,0,0,0,1,0,1

IS7,IS,0,0,0,0,0,0,0,0,0,0,0,0,0,1,1,1,0,1,0,0,0,0,0,0,0,0,0,0,0,0,0,0,1,1,1,0,0,0,0,0,1,0,1,0,0,0,0,0,0,0,0,0,0,0,0,0,0,1,1,1,0,0,0,0,0,0,1,0,0,1,0,1

IS8,IS,0,0,0,0,0,0,0,0,0,0,0,0,0,1,1,0,0,1,0,0,0,0,0,0,0,0,0,1,1,0,0,1,1,1,1,0,0,0,0,0,0,1,0,0,0,0,0,0,0,0,0,1,1,0,0,1,1,1,1,0,0,0,0,1,0,0,1,0,1

Table S5. Primers details.

Primer name	Amplified region	Annealing temperature	Fragment size	Reference
ITS-u1 ǀ ITS-u2	ITS2 (mitochondrial)	55°C	450bp	Cheng et al. 2016
ucp-e ǀ ucp-f/trnF-r	trnL exon2 (plastidial)	55°C	430bp	Taberlet et al. 1991
psbB-B61 F ǀ psbB-B66 R	psbB (plastidial)	45°C	1084bp	Graham and Olmstead 2000
psbC-f ǀ psbC-r	psbC (plastidial)	62°C	865bp	Demesure et al. 1995
SCOT11	N.A.	55°C	N.A.	Collard and Mackill 2009
SCOT12	N.A.	55°C	N.A.	Collard and Mackill 2009
SCOT14	N.A.	55°C	N.A.	Collard and Mackill 2009
SCOT15	N.A.	55°C	N.A.	Collard and Mackill 2009
SCOT16	N.A.	55°C	N.A.	Collard and Mackill 2009

References

Cheng T, Xu C, Lei L, et al (2016) Barcoding the kingdom Plantae: New PCR primers for ITS regions of plants with improved universality and specificity. Mol Ecol Resour 16:138–149. https://doi.org/10.1111/1755-0998.12438

Collard BCY, Mackill DJ (2009) Start Codon Targeted (SCoT) Polymorphism: A Simple, Novel DNA Marker Technique for Generating Gene-Targeted Markers in Plants. Plant Mol Biol Report 27:86–93. https://doi.org/10.1007/s11105-008-0060-5

Demesure B, Sodzi N, Petit RJ (1995) A set of universal primers for amplification of polymorphic non-coding regions of mitochondrial and chloroplast DNA in plants. Mol Ecol 4:129–134. https://doi.org/10.1111/j.1365-294x.1995.tb00201.x

Olmstead RG, Sweere JA (1994) Combining Data in Phylogenetic Systematics: An Empirical Approach Using Three Molecular Data Sets in the Solanaceae. Syst Biol 43:467. https://doi.org/10.2307/2413546

Taberlet P, Gielly L, Pautou G, Bouvet J (1991) Universal primers for amplification of three non-coding regions of chloroplast DNA. Plant Mol Biol 17:1105–1109. https://doi.org/10.1007/BF00037152

SUPPLEMENTARY FIGURES

Figure S1. Genotype Accumulation Curve: A plateau has been reached, then the number of loci is enough to discriminate individuals. Each locus was randomly samples 1000 times to create the distribution.

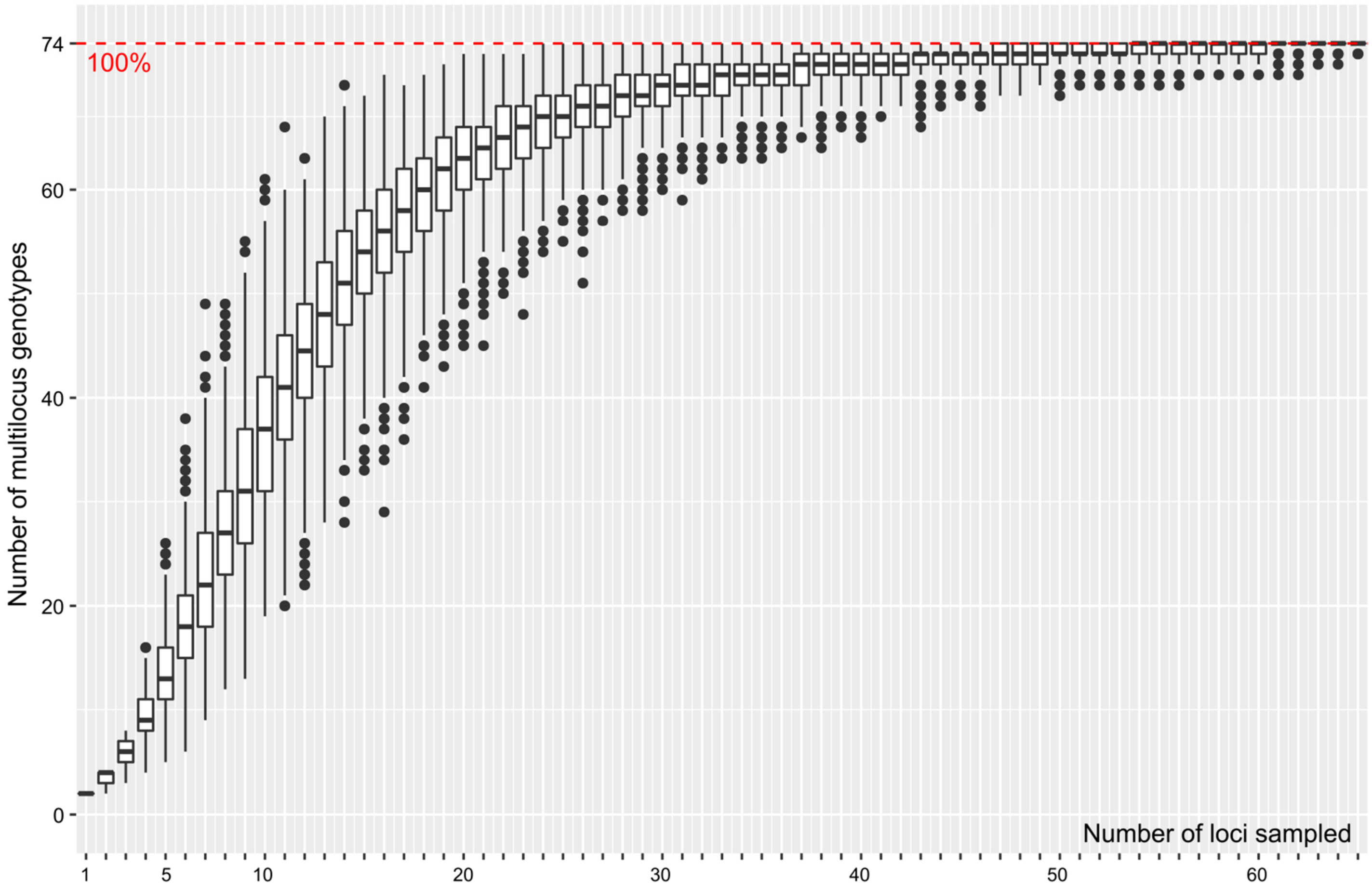

Figure S2. ITS based phylogeny of *Tephroseris* genus considering all the populations: Each label (where available) include the following information: population code, GenBank accession number, species name and ribotype. Labels are colored by geographic origin: T.E.-App = Tosco-Emilian Appennine; Lig.-App = Ligurian Appennine: W-Alps = Western Alps; E-Alps = Eastern Alps; Pannonia = Pannonia geographic region; W-Carp. = Western Carpathians; others = geographic regions far from the study area such as Spain or Turkey. The phylogenetic tree edited int iTOL. The full availability of this figure is available at the link http://itol.embl.de/shared/MartinoAdamo.

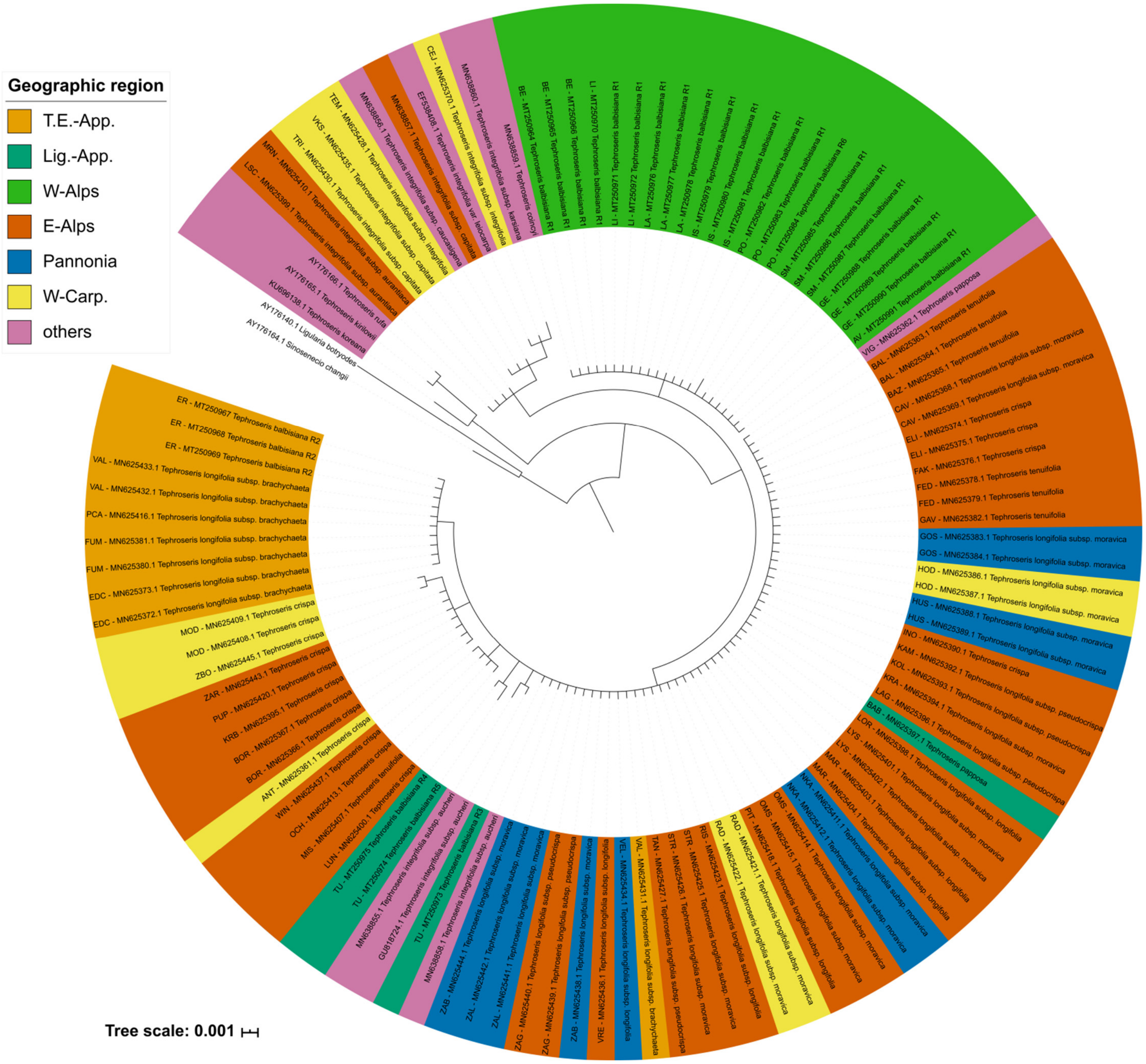

Figure S3. Collinearity among predictors: Predictors used for modelling the species distribution; from top left to bottom right: altitude, isothermality, organic soil content at 15cm depth, average annual precipitations, soil pH at a depth 15 cm, precipitation seasonality, solar radiation, mean annual temperature, annual and daily temperature range, water vapour pressure, wind speed. For each predictor, histograms showing the distribution of extracted values are displayed on the diagonal. Bivariate scatter plots are displayed below the diagonal and the Pearson *r* correlations above the diagonal (threshold for collinearity set at |*r*|> 0.7).

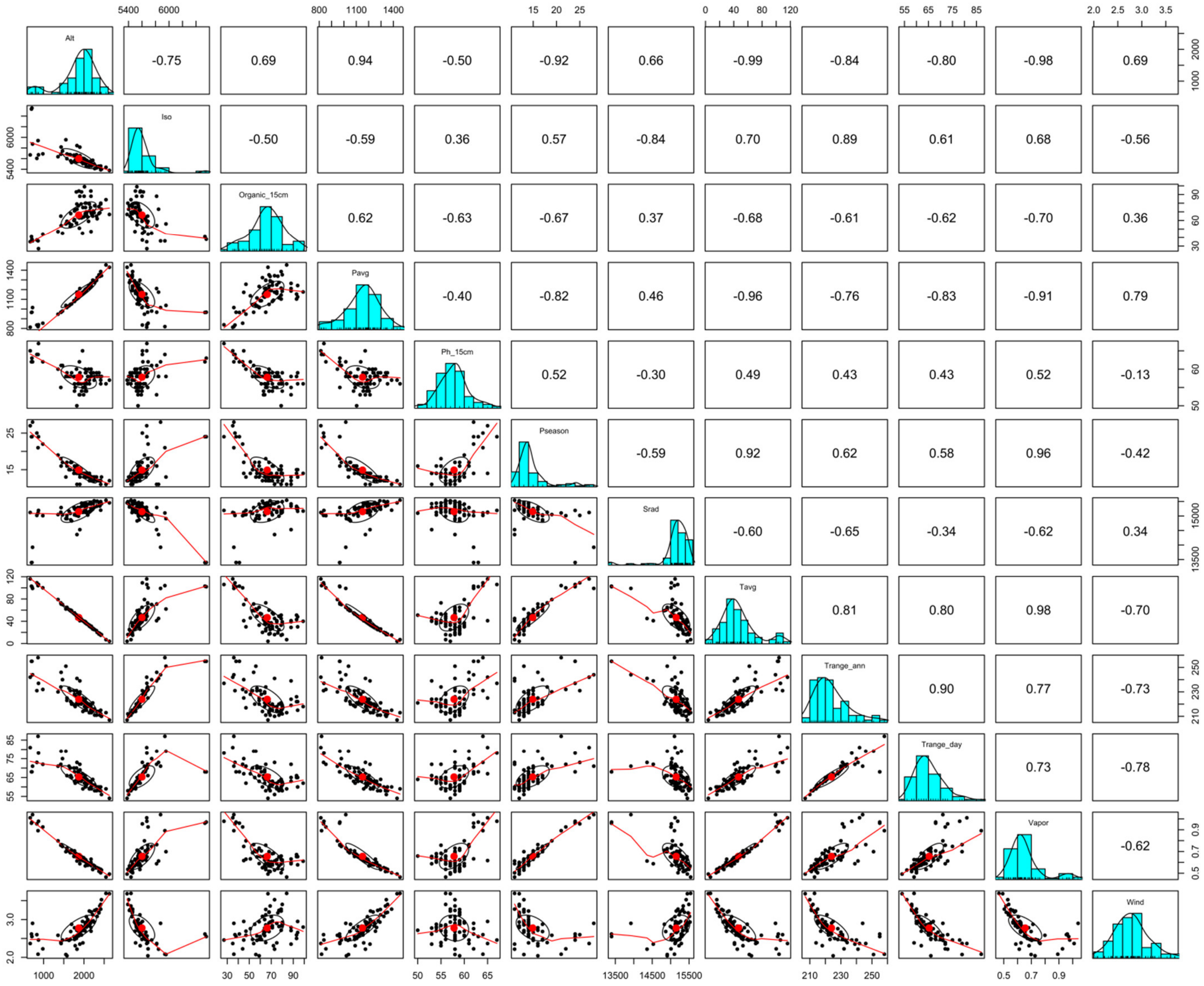

Figure S4. Contribution of variables to principal components (PCs): first ten most important variables, used to measure leaves morphology, are reported in order of relative contribution to PC1 (top graph) and PC2 (bottom graph).

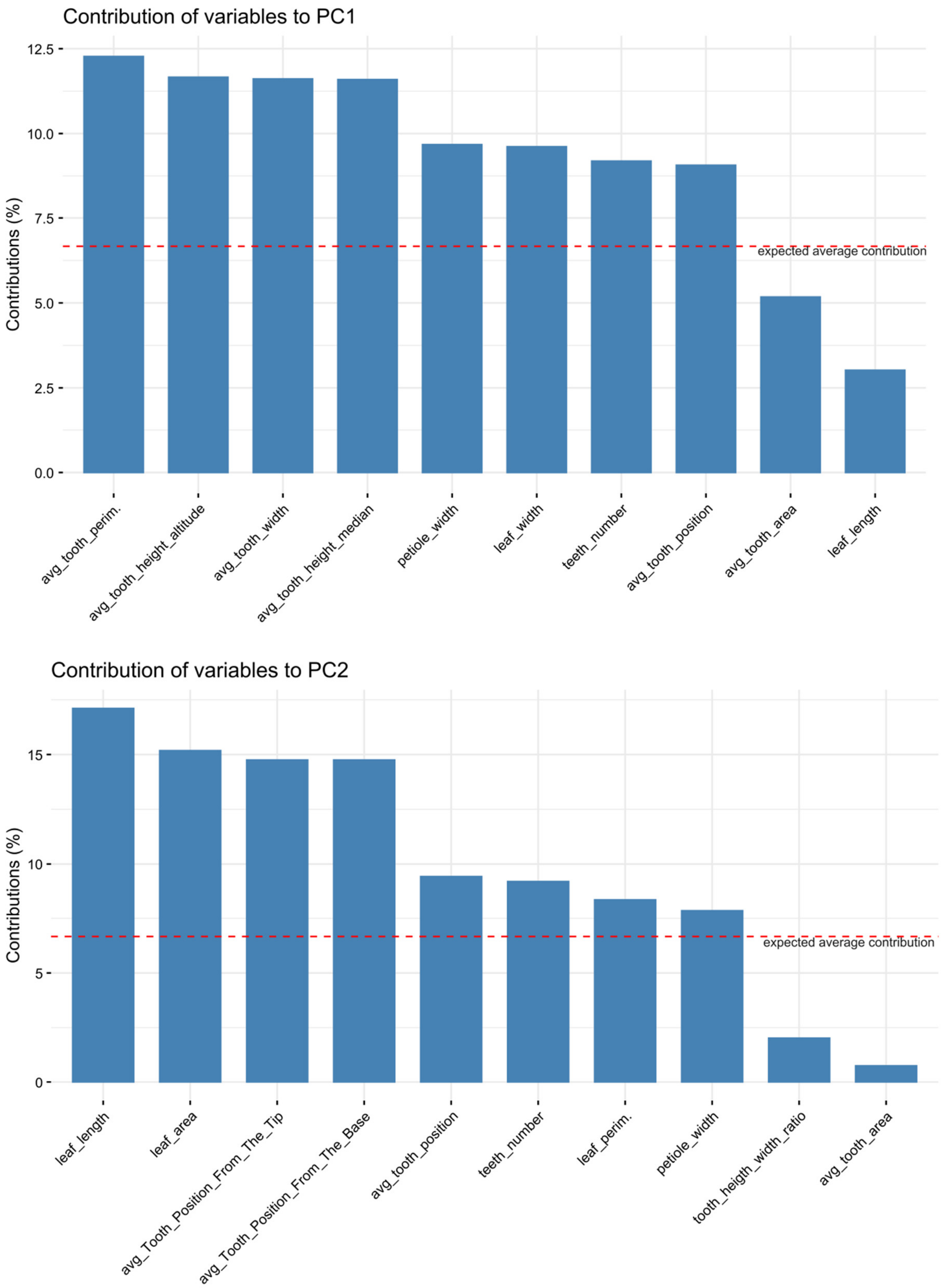

Supplement: Supplementary file 1 [file plants-09-01160-s001.pdf]
